# Doping-Free Phosphorescent and Thermally Activated Delayed Fluorescent Organic Light-Emitting Diodes with an Ultra-Thin Emission Layer

**DOI:** 10.3390/nano13162366

**Published:** 2023-08-18

**Authors:** Eun-Bi Jang, Geun-Su Choi, Eun-Jeong Bae, Byeong-Kwon Ju, Young-Wook Park

**Affiliations:** 1 Nano and Organic-Electronics Laboratory, SunMoon University, Asan 31460, Republic of Korea; kksk0428@sunmoon.ac.kr (E.-B.J.); crs4964@korea.ac.kr (G.-S.C.);; 2 Display and Nanosystem Laboratory, Department of Electrical Engineering, Korea University, 145, Anam-ro, Seoul 02841, Republic of Korea

**Keywords:** organic light-emitting diodes, ultra-thin emissive layer, phosphorescent, thermally activated delayed fluorescence

## Abstract

We report the electroluminescence (EL) characteristics of blue ultra-thin emissive layer (U-EML) phosphorescent (PH) organic light-emitting diodes (OLED) and thermally activated delayed fluorescence (TADF) OLED. A variety of transport layer (TL) materials were used in the fabricated OLEDs. The well-known FIrpic and DMAC-DPS were used with a thickness of 0.3 nm, which is relatively thicker than the optimal thickness (0.15 nm) of the blue phosphorescent ultra-thin emissive layer to ensure sufficient energy transfer. While FIrpic showed overall high efficiency in various TLs, DMAC-DPS exhibited three times lower efficiency in limited TLs. To clarify/identify low efficiency and to improve the EL, the thickness of DMAC-DPS was varied. A significantly higher and comparable efficiency was observed with a thickness of 4.5 nm, which is 15 times thicker. This thickness was oriented from the TADF itself, which reduces quenching in a triplet–triplet annihilation compared to the PH process. The thinner optimal thickness compared with ~30 nm of fluorescent OLEDs suggests that there still is quenching taking place. We expect that the efficiency of TADF U-EML OLEDs can be enhanced through further research on controlling the exciton quenching using multiple U-EMLs with spacers and a novel material with a high energy transfer rate (ΔE_S-T_).

## 1. Introduction

Organic light-emitting diodes (OLEDs) are self-emissive devices composed of organic materials stacked between an anode and cathode, including a hole injection layer (HIL), hole transport layer (HTL), emissive layer (EML), electron transport layer (ETL), and electron injection layer (EIL) [1]. OLEDs have attracted significant attention as the next-generation display technology due to their advantages such as fast response time, high contrast ratio, and low power consumption [2,3,4,5,6,7,8]. Continuous research efforts are focused on enhancing device performance, including high efficiency, high brightness, and long lifetime. OLEDs have been developed into various generations based on their emission mechanisms, including first-generation fluorescent, second-generation phosphorescent, third-generation thermally activated delayed fluorescence (TADF), and fourth-generation hyper fluorescence [9,10,11,12,13].

**Figure 1:**
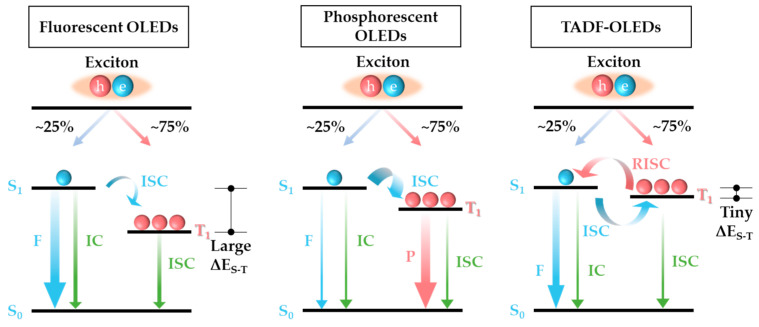
Schematic of electroluminescent exciton dynamic processes of the three generations OLEDs.

Fluorescent OLEDs and phosphorescent (PH) OLEDs are distinguished based on the type of exciton involved. Fluorescence occurs when energy transitions from the excited singlet state (S_1_) to the ground state (S_0_), emitting light in the process. On the other hand, phosphorescence involves energy transition from the excited singlet state to the excited triplet state (T_1_) through intersystem crossing, followed by transition to the ground state, resulting in light emission. PHOLEDs have longer emission times compared to fluorescent OLEDs due to the utilization of both singlet and triplet excitons, with a ratio of 25% singlet and 75% triplet exciton generation based on electron spin arrangement. The comparison diagram of PHOLED and TADF OLED mechanisms is shown in Figure 1. Consequently, phosphorescent materials exhibit higher emission efficiency than fluorescent materials. However, the unstable nature of blue phosphors in phosphorescent OLEDs remains a bottleneck for all phosphorescent OLEDs. The third-generation OLED technology, TADF, utilizes triplet excitons without phosphorescent materials. TADF uses the small energy difference between the singlet and triplet states of materials, allowing the higher-energy triplet state to undergo reverse intersystem crossing (RISC) to the singlet ground state, resulting in delayed fluorescence. While TADF offers the advantage of being capable of fabricating highly efficient fluorescent OLEDs by utilizing both singlet and triplet excitons, it encounters from efficiency roll-off at high brightness due to severe exciton quenching [14,15,16,17,18].

Doping, a commonly used method to improve OLED efficiency, can enhance the electrical and optical properties of devices by facilitating energy transfer between host and guest materials. However, use of multi-doping, such as co-hosts or co-dopants, increases the complexity and cost of equipment and processes. Therefore, an alternative approach has been proposed, which involves inserting an ultra-thin emissive layer (U-EML) with a thickness of less than 1 nm between the charge transport layers to simplify the OLED structure and manufacturing processes [19,20]. U-EML has been studied with various fluorescent, phosphorescent, and TADF materials, demonstrating the realization of not only green, red, and blue OLEDs but also white OLEDs. However, research on U-EML OLEDs using TADF blue materials has not been extensively pursued, calling for further investigations on high-efficiency blue U-EML OLEDs.

In this study, we systematically investigated blue PH and TADF-based U-EML OLEDs to address the increasing demand for high-efficiency blue OLED implementation. We fabricated and compared PH/TADF U-EML OLED devices with different combinations of transport layers, using the representative blue PH/TADF materials, FIrpic and DMAC-DPS. Additionally, we analyzed the absorption/emission spectra and compared the efficiency improvement effects with varying thicknesses of additional emissive materials. Based on these findings, we confirmed the broad compatibility of FIrpic with various transport layers and the limited selectivity of DMAC-DPS. Utilizing this knowledge, we implemented blue U-EML devices based on DMAC-DPS, corresponding to the characteristics of phosphorescent materials. We also explored the characteristics of different U-EML structures arising from differences in the emission principles between PH and TADF materials, highlighting the potential of TADF-based U-EML OLEDs.

## 2. Materials and Methods

### 2.1. Device Structure and Characterization

**Figure 2:**
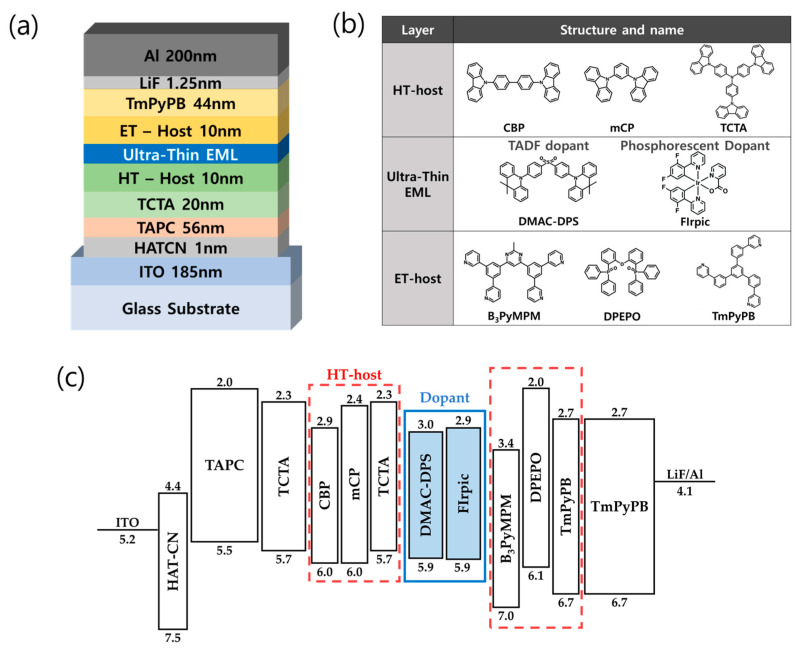
(**a**) Fabricated OLED device structure. (**b**) Molecular structures of the organic materials used in the device. (**c**) Energy level diagram of OLED device with ultra-thin EML structure.

In order to examine and compare the characteristics of ultra-thin emissive layer (U-EML) OLEDs based on various adjacent transport layers, which serve as hosts for the emissive materials, we introduced a phosphorescent (PH) blue U-EML structure [21,22] and kept the basic structure fixed [23]. We compared the characteristics of six devices based on a total thickness of 10 nm (three-hole transport layers (HTL) and three-electron transport layers (ETL)). The device structure, molecular structures, and energy band diagrams are shown in Figure 2.

Figure 2a,b illustrates the device structure, thickness, and materials. The OLED structure is as follows: a 1 nm thick layer of dipyrazino [2,3-f:2′,3′-h]quinoxaline-2,3,6,7,10,11-hexacarbonitrile (HAT-CN) as the hole injection layer, a 56 nm thick layer of di-[4-(N, N-di-p-tolyl-amino)-phenyl]cyclohexane (TAPC) as the hole transport layer, a 20 nm thick layer of 4,4′,4-tris(carbazol-9-yl)triphenylamine (TCTA) as the EML adjacent layer, HTL materials with a thickness of 10 nm, a 0.3 nm thick blue dopant, ETL materials with a thickness of 10 nm, a 44 nm thick layer of 1,3,5-tris(3-pyridyl-3-phenyl)benzene (TmPyPB) as the electron transport layer, lithium fluoride (LiF), and a 200 nm thick layer of aluminum (Al) as the cathode. The HT-host materials, which were deposited with a 10 nm thickness on the HTL interface as the U-EML adjacent layer, included 4,4′-bis(N-carbazolyl)-1,1′-biphenyl (CBP), 1,3-bis(N-carbazolyl)benzene (mCP), and 4,4′,4-tris(carbazol-9-yl)triphenylamine (TCTA). The ET-host materials, which were deposited with a 10 nm thickness on the ETL interface, included 4,6-bis(3,5-di(pyridine-3-yl)phenyl)-2-methyl pyrimidine (B_3_PyMPM), bis [2-(diphenylphosphine)phenyl]ether oxide (DPEPO), and 1,3,5-tris(3-pyridyl-3-phenyl)benzene (TmPyPB). A 0.3 nm thick blue dopant, inserted between the two hosts, consisted of bis [2-(4,6-fluorophenyl) pyridine-C2, N](picolinate)iridium (FIrpic) and 10,10′-(4,4′-sulfonylbis(4,1-phenylene))bis(9,9-dimethyl-9,10-dihydroacridine) (DMAC-DPS). The molecular structures of the HT-host, ET-host, and ultra-thin EML materials used are depicted in Figure 2b.

Figure 2c presents the energy band diagrams of the ultra-thin EML OLEDs used in this study, categorized by dopant. The electrical characteristics of the organic materials used in OLED fabrication, such as highest occupied molecular orbital (HOMO), lowest unoccupied molecular orbital (LUMO), energy bandgap, triplet energy, hole mobility, and electron mobility, are summarized in Table 1.

### 2.2. Experimental Setup and Device Fabrication Methodology

The substrate utilized in this study was indium tin oxide (ITO) coated soda-lime glass, with a sheet resistance of ~18 Ω/sq and a thickness of 185 nm. Cleaning the glass substrates involved the use of an ultrasonic cleaner with acetone, methanol, and deionized water, with each cleaning step lasting 15 min. Subsequently, the cleaned glass substrates were dried for 1 h in a dry oven set at 110 °C. To define the emission area of the OLED, a circular pattern with a diameter of 6.25 mm was created through a photolithography process using a photoresist (AZ-GXR 601, AZ Electronic Materials CO., Ltd., Darmstadt, Germany) on the ITO surface. Surface treatment of the patterned substrates was performed using ultraviolet-ozone (UVC-300, Omniscience, Yongin-si, Korea) and O_2_ plasma (CUTE, Femto Science Co., Hwaseong-si, Korea), aiming to eliminate residues and modify the work function to reduce the operating voltage.

All organic materials and metals used in this study were deposited under high vacuum conditions (~1.0 × 10^−7^ Torr) and with the substrate rotating at a constant speed of 12 rpm during the deposition process. The deposition rates for the organic materials and metals were controlled at a maximum of 1 Å/s and 3 Å/s, respectively. In particular, for the U-EML emissive materials, the deposition rates were adjusted according to thickness to ensure reproducibility and uniformity. For example, for a thickness of 0.15 nm, the deposition was carried out at a rate of approximately 0.015 Å/s, taking approximately 100 s to complete. The thickness of all layers was monitored using a 6 MHz gold-coated quartz crystal microbalance (QCM) and a thin-film deposition controller with a PCI Express interface (IQM-233, INFICON Co., Ltd., Bad Ragaz, Switzerland). The accurate measurement of average thickness was carried out based on the characteristics and composition of organic materials and the deposition process of these thin films follows the island growth concept. Additionally, thin films composed of carbon and iridium exhibit average thicknesses spanning from a few angstroms to tens of angstroms, consistent with the scale observed in various studies.

### 2.3. Characterization and Measurement

The materials’ emission spectra were measured using a fluorescence spectrophotometer (F-7000, Hitachi High-Tech Korea Co., Ltd., Seongnam-si, Korea), and their absorption spectra were obtained through a UV-Vis spectrometer (HP 8453, Agilent Technologies, Inc., Santa Clara, CA, USA). We evaluated the electroluminescent characteristics of the fabricated OLEDs, including their current density-voltage-luminance (J-V-L) behaviors. Before conducting the measurements, the OLED devices were transferred from the glove box to the measurement chamber (in a low-vacuum environment of around 10^−3^ Torr) using desiccants for humidity minimization. This assessment was conducted using a spectroradiometer (CS-2000, Konica Minolta Co., Ltd., Tokyo, Japan) and a source meter (Keithley-2410, Tektronix, Inc., Beaverton, OR, USA). For the OLEDs’ EL characteristics, encompassing current efficiency (CE), power efficiency (PE), and external quantum efficiency (EQE), we measured them vertically, assuming a Lambertian light source. Subsequently, we adjusted the PE and EQE values to consider the emission characteristics measured from specific viewing angles.

## 3. Results

### 3.1. Analysis of Compatibility between TL Hosts According to Dopant

**Figure 3:**
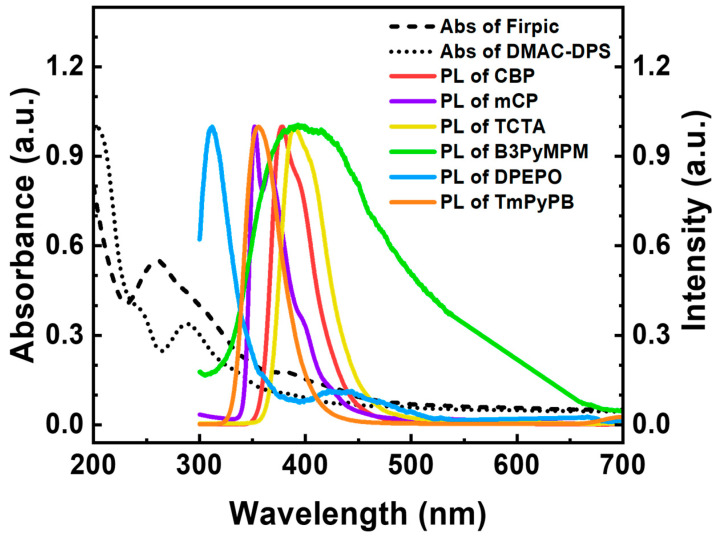
Normalized UV-vis absorption (Abs) and photoluminescence (PL) spectra of emissive and transport materials.

Figure 3 shows the UV-Vis absorption spectra of the emissive materials and the UV-vis photoluminescence (PL) spectra of the transport layer (TL). In the UV absorption spectra, DMAC-DPS exhibits relatively low absorbance and a secondary peak at 288 nm, while FIrpic shows relatively high absorbance and a secondary peak at 261 nm. In the PL spectra, the peak wavelengths were observed in the following order: DPEPO (312 nm), mCP (352 nm), TmPyPB (355 nm), CBP (378 nm), B_3_PyMPM (387 nm), and TCTA (390 nm). Therefore, considering its high absorbance, FIrpic is expected to demonstrate efficient energy transfer, and the choice of TLs may vary due to the differences in secondary peak characteristics.

**Figure 4:**
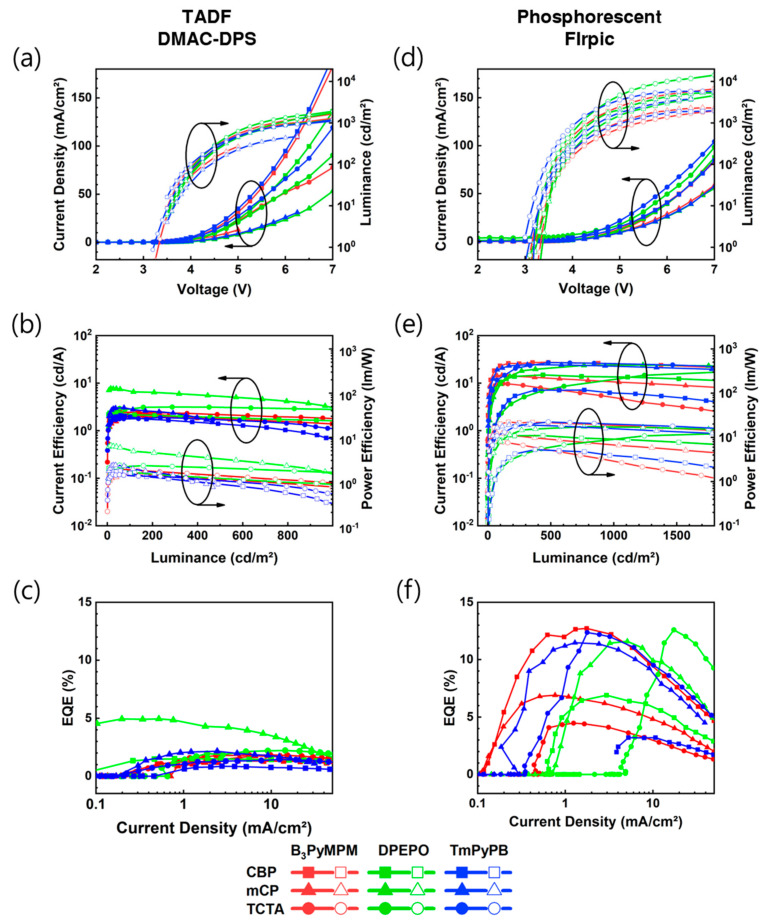
EL characteristics of devices: (**a**,**d**) Current density-voltage-luminance (J-V-L) characteristics. (**b**,**e**) Current efficiency-luminance-power efficiency (CE-L-PE) characteristics. (**c**,**f**) current density–EQE (J–EQE) characteristics.

The EL characteristics of U-EML OLEDs are shown in Figure 4. The fabricated devices utilized the optimal structure for phosphorescent U-EML [36]. The thicker thickness (0.3 nm) was employed, exceeding the optimal thickness (0.15 nm) based on FIrpic, to analyze the optimal energy transfer efficiency between TLs and emissive materials. The peak EQE appeared to be relatively lower compared to previous studies (~18.1%, with TCTA/TmPyPB TLs), as the goal was not to achieve maximum efficiency. When comparing DMAC-DPS (Figure 4a–c) and FIrpic (Figure 4d–f), FIrpic consistently exhibited higher efficiency across various combinations, while DMAC-DPS showed high performance only in specific combinations. This observation indicates that FIrpic’s broad absorbance spectrum contributes to enhanced energy transfer, resulting in a wide range of host selectivity, as depicted in Figure 3. The characteristics of the fabricated devices are summarized in Table 2 and Table 3.

When examining specific details, DMAC-DPS showed a peak EQE of approximately 4.9% specifically with mCP-DPEPO, while other combinations with adjacent TL materials varied from a minimum of two-fold to a maximum of five-fold difference. In contrast, FIrpic achieved high efficiency close to peak EQE regardless of the combination of TL materials, with CBP-B_3_PyMPM (12.7%), mCP-DPEPO (11.6%), mCP-TmPyPB (12.6%), TCTA-DPEPO (11.5%), and TCTA-TmPyPB (12.4%) exhibiting the highest efficiencies. The difference between the highest EQE and peak EQE remained within 10%.

When examining the J-EQE characteristics of FIrpic, variations in the TLs used resulted in different current densities at which the maximum efficiency was achieved. This observation suggests that the influence of energy band and charge mobility, as shown in Figure 2, contributed to these differences. In the case of TCTA/B_3_PyMPM, the high electron-injection barrier of TCTA restricted electron injection, indicating that TCTA-FIrpic energy transfer served as the primary luminescence mechanism. Additionally, in the case of mCP/TmPyPB, due to the relatively high hole mobility, exciton formation at the mCP/TmPyPB interface was hindered and instead occurred at interfaces near high current densities, indicating the influence of a high hole-injection environment. A more detailed analysis (including the sensing layer, etc.) is deemed necessary to confirm these findings.

The maximum EQE of DMAC-DPS devices (4.9%) and FIrpic devices (12.7%) exhibited a significant difference, approximately 2.6-fold. Even when comparing mCP-DPEPO, which is the same TL, the difference remains large at approximately 2.4-fold (4.9%, 11.6%). This difference appears to be primarily influenced by the variation in energy transfer efficiency evident in UV-PL, but considering the molecular distances calculated at a 0.3 nm emissive layer thickness (FIrpic: ~1.09 nm, DMAC-DPS: ~1.02 nm), the occurrence of triplet–triplet annihilation (TTA) is possible. However, considering the difference in luminescence mechanisms and the close match between the secondary absorption peak of DMAC-DPS and the PL of mCP-DPEPO, additional analysis is necessary.

Furthermore, by pursuing optimization for each TL, it appears possible to achieve the highest efficiency within specific current density ranges for FIrpic. Conversely, in the case of DMAC-DPS, efficiency enhancement is attainable specifically with mCP-DPEPO, where the maximum efficiency was observed. Therefore, additional analysis is needed to further understand the luminescence mechanisms of DMAC-DPS and FIrpic, as well as to explore OLEDs incorporating DMAC-DPS U-EML with various thicknesses, not limited to the optimal thickness based on FIrpic.

**Figure 5:**
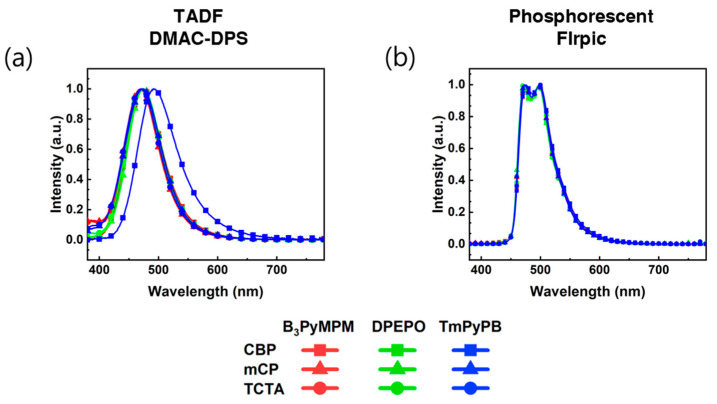
EL Spectra at a current Density of 10 mA/cm^2^: Comparison of Devices Based on Emission Layers Inserted Between HTL and ETL. (**a**) DMAC-DPS, and (**b**) FIrpic.

Figure 5 shows the EL spectra of the fabricated device measured at a current density of 10 mA/cm^2^. Notably, this figure provides a comprehensive comparison of devices based on their emission layers, which are positioned between the HTL and ETL. The EL spectrum for DMAC-DPS is showed in Figure 5a, accompanied by an explanatory description. Additionally, the corresponding spectrum for FIrpic is showcased in Figure 5b, along with a corresponding explanation for comparison purposes. Compared to FIrpic, the DMAC-DPS shows a shifted spectrum with TCTA/B_3_PyMPM. This shifted spectrum is an exciplex emission of TCTA/B_3_PyMPM [37]. The red-shifted and greenish color exciplex state of TCTA/B_3_PyMPM indicates the DMAC-DPS with TCTA/B_3_PyMPM is not suitable for blue OLEDs.

### 3.2. Analysis of EL Characteristics Depending on DMAC-DPS U-EML Thickness Variation

**Figure 6:**
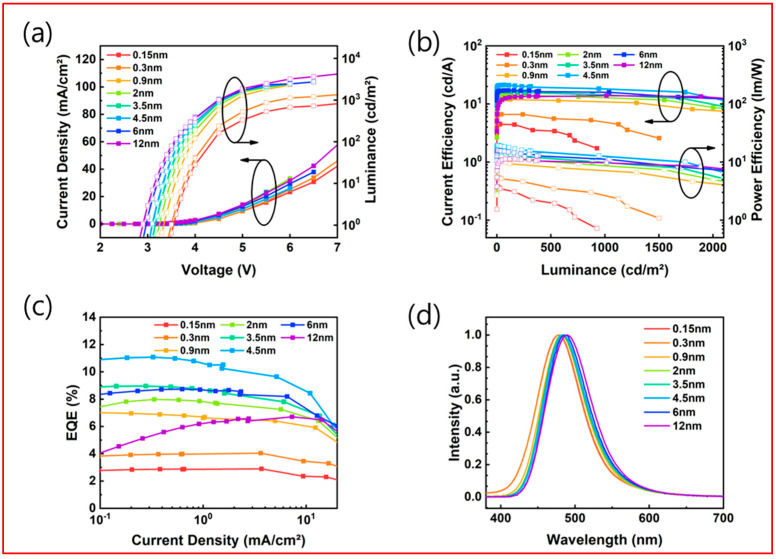
EL characteristics of devices: (**a**) Current density-voltage-luminance (J-V-L) characteristics. (**b**) Current efficiency-luminance-power efficiency (CE-L-PE) characteristics. (**c**) current density–EQE (J–EQE) characteristics. (**d**) Normalized EL spectra of devices.

Figure 6 represents the EL characteristics when adjusting the U-EML thickness of DMAC-DPS. The DMAC-DPS thickness was varied from 0.15 nm to 12 nm, resulting in the fabrication of a total of eight devices. When calculating the average distance between molecules, a thickness of 0.15 nm corresponds to an average distance of 2.1 nm [36], and it is inferred that stacking occurs at distances below the estimated molecular size of 1.3 nm. As shown in Figure 6b,c, the maximum EQE of 11.1% was achieved at a thickness of 4.5 nm and the characteristics of the fabricated devices are summarized in the Table 4.

The implications of the above results are clearly evident. The device efficiency of DMAC-DPS exhibits an initial enhancement followed by a decline as its thickness increases. Notably, the 4.5 nm DMAC-DPS device demonstrates superior light efficiency compared to other devices. Additionally, an increase in DMAC-DPS thickness leads to a decrease in the turn-on voltage. This phenomenon is attributed to the transition from an island-shaped to a film-shaped morphology in the EML, which reduces the occurrence of film defects. The reduction in film defects effectively suppresses leakage current and promotes the movement and recombination of charge carriers. Furthermore, this smooth morphology aids in the efficient transport and recombination of electrons and holes within the EML [38]. The highest efficiency was achieved at a thickness of 4.5 nm DMAC-DPS, where the molecular distance is narrowed to form a complete film. This phenomenon can be attributed to the luminescence principle of TADF. Despite the relatively low energy transfer efficiency, a thickness of 0.3 nm exhibited an efficiency approximately 2.6 times lower than that of FIrpic. However, at a thickness where the efficiency of the emissive material significantly deteriorates, such as 4.5 nm in this case, the maximum efficiency was observed. Generally, for emissive materials, when the molecular distance is narrowed to within 3 nm, TTA and triplet–polaron annihilation (TPA) quenching occur between the triplet excitons, resulting in efficiency degradation. On the other hand, TADF materials exhibit a very narrow energy-level difference between singlet and triplet excitons, enabling triplets to transition to singlets through thermal upconversion. Therefore, TADF materials have a relatively higher likelihood of luminescence before triple–triplet annihilation occurs. Consequently, it was determined that the maximum efficiency at a thickness of 4.5 nm is due to the negligible impact of triple–triplet annihilation. However, it is important to note that this thickness corresponds to a much thinner layer compared to the optimal thickness of approximately 30 nm used in fluorescence devices [39]. The transient decay lifetime (τ) of delayed fluorescence in the case of DMAC-DPS is estimated to be approximately 1.1 μs, attributed to a slower emission rate compared to the nanosecond range of fluorescence [40]. This can be attributed to the slower emission rate in the microsecond range, which is still slower than the nanosecond emission rate in fluorescent materials [41], resulting in increased quenching with increasing thickness. In Figure 6d, the EL spectra exhibit a red shift as the U-EML thickness increases. The thicker the EML, the greater the red shift. This suggests that molecular interaction increases as islands form a film. A DMAC-DPS thickness of 4.5 nm in the mCP-DPEPO TL structure exhibited an EQE of 11.1%, comparable to the 11.6% EQE of 0.3 nm FIrpic. Successful implementation of DMAC-DPS-based U-EML blue OLEDs was achieved and further research incorporating spacer structures or multi-insertion techniques in U-EML fabrication could potentially lead to higher efficiency. Moreover, the utilization of novel materials with smaller ΔE_S-T_ could enable the realization of high-efficiency U-EML TADF blue OLEDs.

## 4. Conclusions

This paper presents a comprehensive investigation into achieving high-efficiency blue OLEDs with a U-EML thickness of less than 1 nm, focusing on TADF and phosphorescent materials. The EL characteristics were systematically investigated based on the materials used for adjacent charge transport layers. DMAC-DPS, a TADF emissive material, and FIrpic, a phosphorescent emissive material, were evaluated with various commonly used charge transport layers (CBP, mCP, TCTA) and electron transport layers (B_3_PyMPM, DPEPO, TmPyPB). The results revealed significantly different effects of adjacent TLs on ultra-thin blue EML performance.

FIrpic consistently achieves nearly identical maximum EQE across different adjacent transport layers, with variations of less than 10% compared to the highest EQE. In contrast, DMAC-DPS exhibits peak EQE differences of up to five times depending on the adjacent transport layer. While FIrpic’s performance is significantly influenced by charge balance between materials, additional engineering of charge transport or injection layers can enhance power efficiency. FIrpic is suitable for high-efficiency ultra-thin EML blue OLEDs.

Furthermore, we analyze the distinct characteristics resulting from the unique TADF mechanism of DMAC-DPS by varying its thickness. The maximum EQE was obtained at a thickness 30 times thicker than the optimized condition for phosphorescent emission. This result was attributed to the difference in emission mechanisms between phosphorescence and TADF. Even at the thicker thickness where the phosphorescent material was fully quenched due to a lower TTA rate in DMAC-DPS, high efficiency was achieved, indicating that the maximum efficiency was achieved at a stacking thickness of 4.5 nm, where the intermolecular distance was zero. This suggests the possibility of achieving higher efficiency through the application of TADF materials with minimized ΔE_S-T_ and the improvement of efficiency in thicker emissive layers through spacer-EML multilayer structures.

In conclusion, this comparative study on ultra-thin EML OLEDs based on phosphorescent/TADF OLEDs for achieving high-efficiency blue OLEDs successfully demonstrated the influence of adjacent transport layers on ultra-thin blue EML performance. It was confirmed that DMAC-DPS, a TADF blue material, exhibits much higher material dependence compared to FIrpic, a phosphorescent blue material. Moreover, FIrpic as a phosphorescent material showed highly desirable properties for implementing ultra-thin EML blue OLEDs, while DMAC-DPS TADF OLEDs achieved high efficiency even at relatively thicker thicknesses. This study provides a foundation for further research in achieving high-efficiency U-EML OLEDs.

## Figures and Tables

**Table 1:** Summarized photophysical and electrochemical characteristics of organic materials in OLED fabrication.

Material	LUMO [eV]	HOMO [eV]	E_g_^1^ [eV]	E_T_ ^2^ [eV]	μ_h_ ^3^ [cm^2^ V^−1^ s^−1^]	μ_e_^4^ [cm^2^ V^−1^ s^−1^]	Function	Reference
HAT-CN	4.4	7.5	3.1	-	-	-	HIL	[24]
TAPC	2	5.5	3.5	2.87	1 × 10^−3^	5.0 × 10^−6^	HTL	[25,26]
CBP	2.9	6	3.1	2.6	3 × 10^−4^	1.0 × 10^−8^	HTL	[25,27,28]
mCP	2.4	6	3.6	2.9	1.2 × 10^−4^	5.0 × 10^−6^	HTL	[25,29]
TCTA	2.3	5.7	3.4	2.79	3 × 10^−3^	1.0 × 10^−8^	HTL	[27,28,30]
DMAC-DPS	2.9	5.9	3	2.91	-	-	TADF dopant	[31]
FIrpic	3	5.9	2.9	2.65	-	-	PH dopant	[19]
B_3_PyMPM	3.4	7	3.6	3.08		4.0 × 10^−3^	HTL	[32,33]
DPEPO	2	6.1	4.1	3	1 × 10^−9^	5.62 × 10^−6^	HTL	[31]
TmPyPB	2.7	6.7	4	2.75	5.0 × 10^−6^	1.0 × 10^−3^	HTL	[34]
LiF/Al	4.1	4.1	-	-	-	-	EIL	[35]

^1^ Band gap energy. ^2^ Triplet energy. ^3^ Hole mobility. ^4^ Electron mobility.

**Table 2:** Summary of device characteristics for DMAC-DPS emissive material.

Emissive Materials: DMAC-DPS		EQE [%]		CE [cd/A]		PE [lm/W]	
HTL	ETL	Peak	@100 cd/m^2^	Peak	@100 cd/m^2^	Peak	@100 cd/m^2^
CBP	B_3_PyMPM	1.3	1.3	2.1	2.1	1.8	1.6
	DPEPO	1.7	1.6	2.2	2.2	1.6	1.6
	TmPyPB	1.8	1.8	2.3	2.3	1.8	1.7
mCP	B_3_PyMPM	1.5	1.5	2.4	2.2	2.1	1.6
	DPEPO	4.9	4.3	7.7	6.6	5.8	4.8
	TmPyPB	2.2	1.9	3.1	2.8	2.4	2.2
TCTA	B_3_PyMPM	0.9	0.8	2.0	1.9	1.7	1.5
	DPEPO	2.1	2.0	3.0	2.8	2.3	1.9
	TmPyPB	1.5	1.3	1.9	1.8	1.5	1.4

**Table 3:** Summary of device characteristics for FIrpic emissive material.

Emissive Materials: FIrpic		EQE [%]		CE [cd/A]		PE [lm/W]	
HTL	ETL	Peak	@100 cd/m^2^	Peak	@100 cd/m^2^	Peak	@100 cd/m^2^
CBP	B_3_PyMPM	12.7	12.2	9.0	9.4	7.9	7.8
	DPEPO	6.9	4.8	14.0	14.6	12.2	12.1
	TmPyPB	4.5	2.1	27.3	26.2	22.1	19.4
mCP	B_3_PyMPM	6.9	6.2	14.8	13.4	11.0	8.9
	DPEPO	11.6	11.6	24.1	24.1	16.8	16.8
	TmPyPB	12.6	6.9	26.7	14.6	16.8	10.8
TCTA	B_3_PyMPM	3.2	2.7	7.2	5.9	5.3	3.5
	DPEPO	11.5	10.9	24.3	23.0	19.1	16.1
	TmPyPB	12.4	12.0	27.0	26.2	22.3	20.5

**Table 4:** Summary of DMAC-DPS based U-EML OLED device characteristics at varied thicknesses.

Thickness of U-EML [nm]	V_on_^1^ [V]	EQE [%]		CE [cd/A]		PE [lm/W]	
		Peak	@500 cd/m^2^	Peak	@500 cd/m^2^	Peak	@500 cd/m^2^
0.15	3.6	2.9	2.3	4.5	3.4	3.8	1.9
0.3	3.5	4.0	3.5	6.6	5.6	5.5	3.5
0.9	3.3	7.0	6.4	12.9	11.4	11.9	8.0
2.0	3.3	8.2	7.3	14.8	13.3	12.7	9.3
3.5	3.2	9.0	7.8	16.9	14.6	15.7	10.2
4.5	3.1	11.1	10.5	21.2	20.0	19.4	15.7
6.0	3.0	8.7	8.6	17.0	16.7	13.1	13.1
12.0	2.9	6.7	6.6	13.9	13.7	11.3	10.8

^1^ Turn-on voltage (V_on_).

## Data Availability

Not applicable.
